# Al_2_O_3_-Coated Si-Alloy Prepared by Atomic Layer Deposition as Anodes for Lithium-Ion Batteries

**DOI:** 10.3390/ma15124189

**Published:** 2022-06-13

**Authors:** Kikang Lee, Sungho Yoon, Sunghoon Hong, Hyunmi Kim, Kyuhwan Oh, Jeongtak Moon

**Affiliations:** 1 Research and Development Center, MK Electron, Yongin-si 17030, Korea; kikang86@snu.ac.kr (K.L.); hongsh@mke.co.kr (S.H.); 2 Department of Materials Science and Engineering, Seoul National University, Seoul-si 08826, Korea; kyuhwan@snu.ac.kr; 3 Electronic Convergence Materials and Device Research Center, Korea Electronics Technology Institute (KETI), Seongnam-si 13509, Korea; 36204@keti.re.kr (S.Y.); hyunmi@keti.re.kr (H.K.); 4 Department of Mechanical Engineering, Sungkyunkwan University, Suwon-si 16419, Korea

**Keywords:** anode materials, lithium-ion battery, atomic layer deposition, silicon nanocomposite, Al_2_O_3_ layer, single layer pouch cell

## Abstract

Silicon-based anodes can increase the energy density of Li-ion batteries (LIBs) owing to their large weights and volumetric capacities. However, repeated charging and discharging can rapidly deteriorate the electrochemical properties because of a large volume change in the electrode. In this study, a commercial Fe-Si powder was coated with Al_2_O_3_ layers of different thicknesses via atomic layer deposition (ALD) to prevent the volume expansion of Si and suppress the formation of crack-induced solid electrolyte interfaces. The Al_2_O_3_ content was controlled by adjusting the trimethyl aluminum exposure time, and higher Al_2_O_3_ contents significantly improved the electrochemical properties. In 300 cycles, the capacity retention rate of a pouch full-cell containing the fabricated anodes increased from 69.8% to 72.3% and 79.1% depending on the Al_2_O_3_ content. The powder characterization and coin and pouch cell cycle evaluation results confirmed the formation of an Al_2_O_3_ layer on the powder surface. Furthermore, the expansion rate observed during the charging/discharging of the pouch cell indicated that the deposited layer suppressed the powder expansion and improved the cell stability. Thus, the performance of an LIB containing Si-alloy anodes can be improved by coating an ALD-synthesized protective Al_2_O_3_ layer.

## 1. Introduction

Li-ion batteries (LIBs) are widely used in energy-intensive mobile electronic devices, such as smartphones, electric cars, and notebooks, owing to their high energy densities, long cycle lives, and low self-discharge rates. Recently, high-capacity LIBs with high Coulombic efficiency have been developed for electric vehicles and large-scale renewable energy storage systems to reduce the global environmental pollution [1,2,3,4,5].

An LIB is composed of a cathode, anode, and separator. For the last two decades, graphite has been widely used as a standard anode material in rechargeable LIBs owing to its low working potential, structural stability, cost effectiveness, and long cycle life [6,7,8,9].

Although carbon-based anode materials exhibit numerous advantages in addition to being inexpensive and highly stable, their limited capacity (theoretical capacity: 372 mAhg^−1^) is a major drawback that impedes their application in current high-capacity devices. Consequently, efforts to identify suitable alternatives to graphite have gained momentum, and among such materials, Si has emerged as a viable alternative candidate for advanced applications [10,11,12].

Si exhibits a high theoretical specific capacity of 3580 mAhg^−1^, a large weight, superior capacity per volume, and the lowest discharge voltage, and forms the Li1_5_Si_4_ alloy. Thus, Si is the most suitable anode material for high-energy-density LIBs [13,14,15,16,17,18,19,20,21]. However, despite its high capacity, Si is difficult to commercialize. Structurally, a large volume expansion occurs while alloying a metal with Li [22,23,24,25]. The volume of Si can increase/decrease during the charging/discharging process through the formation of an electrochemical alloy with Li. Such volume changes due to charging and discharging generate cracks on the surface of the active electrode material, and continuous cracks can cause micronization of the electrode surface. This micronization through reactions with the electrolyte generates new interfaces, called solid electrolyte interfaces (SEIs). This SEI layer is insulating and causes depletion of the electrolyte, resulting in a rapidly decreasing capacity during the charging/discharging process in a cycle, and this loss of capacity after each cycle degrades the cycle performance in the long run [13,26,27,28,29,30,31].

One method for mitigating this drawback of using Si is to coat the Si-based anode with various materials, which suppress the volume expansion and prevent direct contact with the electrolyte [32,33]. Various methods such as wet coating, physical vapor deposition, chemical vapor deposition (CVD), and atomic layer deposition (ALD) are employed to coat the anode with a suitable material [34,35]. Among them, the wet coating method is relatively simple but has the disadvantage of a low diffusion rate, and the final product may show poor homogeneity according to the mixing energy. Moreover, separating a solid product by removing the solvent is an essential step in the conventional wet coating process. This step may cause the mixing of impurities in the final product as well as increase the cost and complexity [36,37].

ALD is a promising coating method that can be used to deposit highly homogeneous and reproducible materials on a powder surface. This process is an optimized and improved version of the CVD technique. CVD can be used to deposit a thick film easily, because in this method, the surface reaction of the target itself, such as the substrate, is induced by the applied voltage, plasma, or heat after the simultaneous injection of large volumes of gases. In contrast, in ALD, the film is coated through the self-limiting reaction of the precursor molecules on the substrate surface with each precursor gas and reactant gas in alternating pulses. Compared to the CVD method, ALD enables the deposition of a uniform coating on the substrate because, in this case, a single layer is deposited in each pulse step. Another advantage of the ALD process is that the thickness of the coating layer can be finely adjusted by repeating cycles of the ALD process [38,39,40,41,42].

ALD is typically used to deposit a film on a substrate. Recently, research on improving the performance of LIBs using ALD has been actively conducted. In particular, various materials such as TiO_2_, TiN, HfO_2_, Y_2_O_3_, and Al_2_O_3_ have been deposited, at a nanoscale thickness, to improve the performance of electrode and battery powder materials [43]. In this study, ALD was used to coat a layer of Al_2_O_3_ onto the surface of a Si-based anode material powder. The Al_2_O_3_ layer was coated using trimethyl aluminum (TMA), which can be deposited and coated at a relatively low temperature (≤200 °C) and exhibits the advantages of volatility and pyrolysis resistance (<370 °C). This prevents deterioration (Si and iron silicide phase changes) of the commercial Si-based anode powder [44,45,46,47]. Synthesis using TMA and H_2_O is an ideal ALD process that has been widely researched [48].

Herein, a commercially available Fe-Si anode material was used as the anode to improve the electrochemical performance, prevent volume expansion, which causes electrochemical inferiority, and suppress the formation of SEIs due to cracks. Using the ALD process, an Al_2_O_3_ layer was deposited for each condition to form a 30-nm thick protective layer on the powder. The physical properties of the deposited Al_2_O_3_ layers were analyzed using various characterization techniques, including X-ray fluorescence (XRF), particle size analysis, X-ray diffraction (XRD) pattern analysis, Brunauer–Emmett–Teller (BET) surface area analysis, scanning electron microscopy (SEM), and transmission electron microscopy (TEM). Further, the electrochemical properties of the Al_2_O_3_ layer, deposited as an anode material for LIBs, were evaluated using coin and pouch cells. The rate characteristics, cyclic voltage current, as well as the contraction and expansion of the pouch cell during the charging and discharging process were analyzed. The results showed that the ALD-synthesized Al_2_O_3_ coating layer played a key role in improving the electrochemical performance of the cells via powder surface modifications [49].

## 2. Materials and Methods

### 2.1. Experimental

#### 2.1.1. Al_2_O_3_ ALD Procedure

The Si-alloy-based active anode material was sourced from MK Electron Co., Ltd. (MKE, Gyeonggi, Korea) in powder form and is composed of a Fe-Si (15 at.%–85 at.%) alloy melted by vacuum induction. The molten Fe-Si alloy and chrome-steel balls were loaded into the mechanical alloy milling instrument (CM20, ZoZ GmbH, Wenden, Germany) at a ratio of 1:15, and then milled at 650 rpm for 12 hours. The resulting powder was then pulverized into a uniform particle size using an Air Jet Mill (Jet Mill-LB, KM tech, Gyeonggi-do, Korea) under operating conditions of (1) powder feed rate: 1 kg/h, (2) push press: 0.7 mPa, and (3) grind press: 0.4 mPa. Through the above process of mechanical alloying, the molten Fe-Si alloy formed a phase wherein amorphous Si nanoparticles are dispersed in an inert (inactive) iron silicide matrix. The active Si content was gradually decreased to improve the electrochemical performance of the resulting powder. Herein, this primary anode material is referred to as the “N(Nanocrystalline)-Si alloy”.

The ALD process was used to coat a layer of Al_2_O_3_ on the N-Si alloy powder provided by the manufacturer, which had a particle diameter of approximately 3 µm (d = 0.5). A 300 cc rotary-type ALD system with a reactor (Atomic Shell, CN1, Gyeonggi-do, Korea) was used to coat the powder using the ALD method. In this 300 cc tumbler-type cylindrical reactor, 5 g of the N-Si alloy powder and 60 g of 1-mm silica balls, used for the powder dispersion, were charged simultaneously at 150 °C under rotation at 30 rpm. The TMA, N_2_, and H_2_O gases were sequentially injected during the rotation, with a sufficient soaking time after the injection of each gas to allow enough time for the reaction to progress.

Table 1 lists the conditions for injecting the TMA, N_2_, and H_2_O gases to observe the changes in the properties according to the thickness of the ALD-synthesized coating layer. The thickness of the Al_2_O_3_ layer can be increased by repeating the process cycles several times. However, only two cycles were repeated for each condition, as cycle repetition can hinder the passivation of Li ions during the electrochemical evaluation [50]. The Al_2_O_3_-coated samples prepared under each condition are referred to as “Al_2_O_3_-1” and “Al_2_O_3_-2” in this paper.

#### 2.1.2. Material Characterization

The phase and crystallinities of the N-Si alloy powders, coated with Al_2_O_3_ using ALD, were analyzed using XRD (D8, Bruker AXS, Karlsruhe, Germany) with Cu-K_α_ (wavelength: 0.15418 nm). The exposure time was 2.5 s at each step, and the scanning was performed in the range of 2*θ* = 10–60° with an interval of 0.45°. The morphology of the coated N-Si alloy powder was analyzed using field-emission SEM (JSM-6701F, JEOL, Tokyo, Japan) and field emission gun TEM (JEM-ARM200F, JEOL, Japan). Focused Ion Beam (FIB-FB2100, Hitachi, Japan) treatment was performed to observe the cross section of the powder. The compositions of the powders were identified using the XRF technique (ZSX Primus, Rigaku, Japan), and the powder-particle sizes were analyzed using a laser diffraction particle size analyzer (Mastersizer 2000, Malvern, UK). Furthermore, a BET analyzer (BET-Micromeritics ASAP2020, Norcross, GA, USA) was applied to evaluate the specific surface area and pore size distribution of the synthesized coated N-Si alloy powders.

#### 2.1.3. Electrode Preparation

In this study, two types of electrode plates were fabricated to evaluate the electrochemical properties of ALD-synthesized N-Si alloy powders. Anode plates, containing 80 wt.% of the coated N-Si alloy powders, were prepared according to the method discussed in a later section to fabricate coin half-cells (CHCs), which were employed to measure the initial capacity and Coulombic efficiency of the coated N-Si alloy powders. In addition, electrode plates, containing 15 wt.% of the ALD-synthesized N-Si alloy powders, were prepared according to the method presented in a later section to fabricate two different types of cells. Specifically, pouch full-cells (PFCs) were used to evaluate the cycling performance, whereas single-layer pouch cells (SLPCs) were employed to examine cell contraction and expansion.

#### 2.1.4. Preparation of 80 wt.% Fe-Si Electrode for CHCs

The slurry applied to the anode plates was prepared by mixing 80 wt.% of the active materials (N-Si alloy, Al_2_O_3_-1, and Al_2_O_3_-2), 5 wt.% of a conductive agent (Super P, TIMCAL, Tokyo, Japan), and 15 wt.% of polyacrylic acid binder (PAA, AST-9005, Aekyung chemical, Seoul, Korea). First, the PAA binder and conductive agent were mixed for 5 min at a rotation speed of 2000 rpm. Next, the active material was added and mixed for 5 min at 2000 rpm using a Thinky mixer (ARE-310, Thinky, Laguna Hills, CA, USA). Then, distilled water was added to adjust the solid content to ~30 wt.%, and the product was mixed for 10 min at 2000 rpm. The electrode plates were fabricated by setting the blade gap and applying 2.5 mg/cm^2^ of slurry onto a Cu foil (18-μm thick, UACJ, Tokyo, Japan) using the doctor blade technique to form a smooth surface. The electrode plates were then dried at 110 °C for 10 min and punched to form circular shapes with a diameter of 16 mm, followed by rolling to form circular plates electrodes with a volume of 1.5 g/cm^3^. These rolled electrodes were spot-welded to the inner base of a 2032-type coin cell case, and the entire assembly was then vacuum dried at 110 °C for 12 h.

#### 2.1.5. Preparation of 15 wt.% Fe-Si Electrode for PFCs and an SLPC

To evaluate the lifespan and volume expansion behavior of the cells, 15 wt.% of the active materials (N-Si alloy, Al_2_O_3_-1, and Al_2_O_3_-2), 81 wt.% of graphite (95 wt.% of 918(II) (BTR, Shenzhen, China), 5 wt.% of SFG6 (TIMCAL, Japan)), 1.5 wt.% of carboxymethylcellulose (CMC; 350HC, Nippon Paper, Hokkaido, Japan), and 1 wt.% of conductive agent (Super P Li, TIMCAL) were mixed at 2000 rpm for 3 min using the same Thinky mixer. Next, distilled water was added to adjust the solid content to ~60 wt.%, and the product was again mixed at 2000 rpm for 3 min. Finally, 1.5 wt.% of styrene-butadiene rubber (SBR; BM400-B, Zeon, Tokyo, Japan) was added and mixed at 2000 rpm for 3 min. This product was then cast on both sides of a smooth Cu foil (10-μm thick, UACJ, Tokyo, Japan) using the doctor blade technique. The electrode plates were fabricated by adjusting the blade gap such that a 7 mg/cm^2^ layer of the mixed slurry is formed on both sides of the Cu foil. These electrode plates were then dried in air at 110 °C for 10 min and punched to form square-shaped electrodes (dimensions: 59 × 86 and 33 × 84 mm for the PFC and SLPC, respectively) with a gap of 1 cm. These electrodes were subsequently rolled to form circular electrode plates with a density of 1.6 g/cm^3^ and finally dried in a vacuum at 110 °C for 12 h. 

#### 2.1.6. Cell Assembly

Three types of batteries, viz. CHCs, PFCs, and SLPCs, were fabricated. To evaluate the initial capacity and Coulombic efficiency, 2032-coin-cell-type CHCs were used. The moisture and oxygen concentration was controlled to <1 ppm, and the cell was assembled inside a glove box filled with Ar gas (KK-021-AS, Koreakiyon, Seoul, Korea). For the counter electrode, a Li-metal chip with a thickness of 0.45 mm (EQ-Lib-LiC45, MTI, Jeollanam-do, Korea) was used. A separator with a diameter of 17 mm and thickness of 24 μm (polypropylene 3501, Celgard, Japan) was placed between the anode and the Li-metal chip. The CHC electrode was assembled by filling it with 1.0 M LiPF_6_ in an ethylene carbonate (EC)/diethyl carbonate (DEC)/fluoroethylene carbonate (FEC) ratio of 5/70/25 (*v*/*v*).

**Figure 1:**
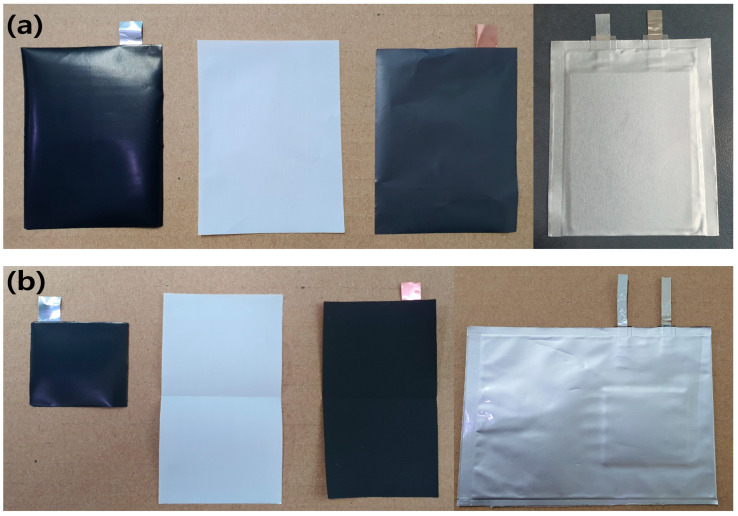
Photographs of the cathode, separator, and anode components (left) and the assembled cells (right) for the (**a**) PFCs and (**b**) SLPCs.

**Figure 2:**
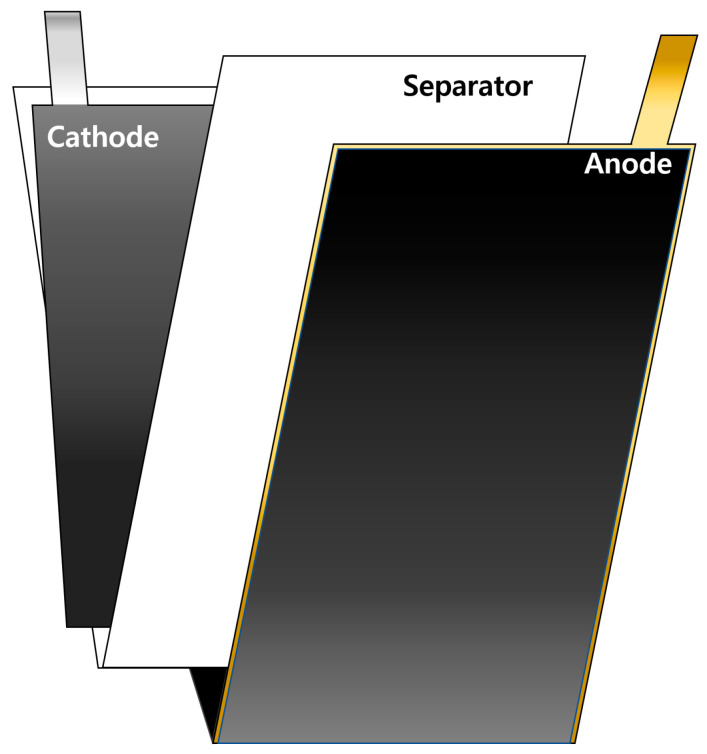
Schematic of the electrode of the SLPC.

To evaluate the lifespan, PFCs were fabricated by stacking three anode sheets and two cathode sheets, all of which were coated on both sides. As shown in Figure 1a and schematically illustrated in Figure 2, the SLPCs were assembled in a dry room using sheets of the anode and cathode, both of which were also coated on both sides. Furthermore, as shown in Figure 1, LiNi/Mn/Co/O_2_ 622 (NCM622) was used as the cathode of both the PFCs and SLPCs. The cathode was a mixture of 94 wt.% NCM622 (Umicore, Brussels, Belgium), 3 wt.% conductive agent (Ketjenblack-EC600JD, Lion Corporation, Sumida-ku, Japan), and 3 wt.% binder (polyvinylidene difluoride, Solvay, Brussels, Belgium). The N/P ratio was set to 1.1:1, and an aerial capacity of 3.0 mAh/cm^2^ was achieved. The Cu and Ni metal strips were welded to the prepared cathode and anode, respectively (cathode: Ni, anode: Cu). The length of these strips for the PFCs and SLPCs were 1 and 3 cm, respectively. After inserting the assemblies into the pouches and filling the pouches with the electrolyte, the excess gas in the cell was removed using a vacuum heat-sealing process. The cell was then assembled using a 24-μm-thick separator (polypropylene 3501, Celgard, Japan), and 1.0 M LiPF_6_ in an EC/DEC/FEC ratio of 25/70/5(*v*/*v*%) was filled as the electrolyte. 

#### 2.1.7. Electrochemical Investigations of the Cells

*CHC setup*—The electrochemical properties of the N-Si alloy, Al_2_O_3_-1, and Al_2_O_3_-2, namely, their initial capacity and Coulombic efficiency, were evaluated using the TOSCAT-3100 (TOYO SYSTEM) battery tester. For evaluating the CHC, charging/discharging was performed at 0.1 C. The charging constant current (CC) cutoff voltage was set to 0.01 V, and the constant voltage (CV) was maintained until 0.01 C. The discharge CC cutoff voltage was set to 1.5 V. 

*PFC setup*—For evaluating the PFC, the voltage was set in the range of 4.2–2.7 V. The formation cycle was conducted with C-rates of 0.05, 0.01, 0., and 0.5 C in the first (charging, discharging), second, third, and fourth cycles, respectively. The charge cutoff was set to 4.2 V for CC and 0.05 C for CV. The discharge CC cutoff was set to 2.7 V. 

To evaluate the lifespan after the formation cycle, the cutoff was set to 0.5 C for the charging and 1 C for discharging phase. The cutoff conditions were the same as those in the formation stage. For the PFCs, 1 C corresponded to a specific current density of 172 mA g^−1^ for the cathode active material NMC622. Furthermore, the output characteristics were evaluated at various C-rates (low-high-low), as shown in Table 2.

*SLPC setup*—To verify the expansion and contraction of the SLPCs, the voltage was set in the range of 4.2–2.7 V. The formation cycle was performed at C-rates of 0.1, 0.2, and 0.5 C in the first (charging, discharging), second, and third cycles, respectively. The charge cutoff was set to 4.2 V for CC and 0.05 C for CV. The discharge cut-off was set to 2.7 V for CC. After the formation cycle, the cycle condition was set to 0.5 C for both the charging and discharging cases. The cutoff conditions were the same as those in the formation stage. The evaluation of the SLPC was performed in a high-temperature chamber at 60 °C.

## 3. Results and Discussion

### 3.1. XRF and Powder-Particle Size Analyses

**Figure 3:**
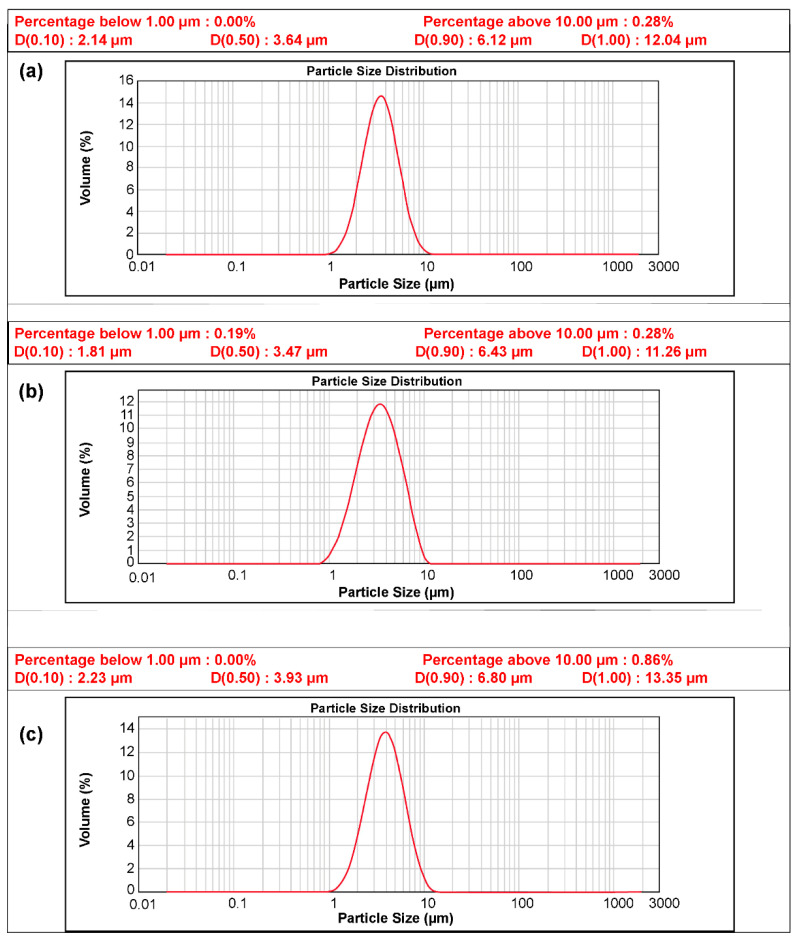
Particle distribution curve of the powders: (**a**) N-Si alloy, (**b**) Al_2_O_3_-1, and (**c**) Al_2_O_3_-2.

The size and composition of the pristine N-Si alloy powder and N-Si alloy powder coated with Al_2_O_3_ were examined using powder-particle size analysis, as shown in Figure 3 and XRF composition analysis (Table 3). The advantage of the ALD process is that the impurity content can be minimized, and a thin layer, which can be dispersed on the powder, can be formed [51]. The average particle size of the N-Si alloy was found to be 3.47 µm, while those of the Al_2_O_3_-1 and Al_2_O_3_-2 samples, prepared using the ALD technique, were 3.64 and 3.93 µm, confirming that ALD increased the particle size. However, the increase in the particle size did not affect the electrodes during the fabrication and can be interpreted as the formation of a homogeneous thin layer. The XRF composition analysis showed that the Al content in the ALD-synthesized Al_2_O_3_-coated samples, viz. Al_2_O_3_-1 and Al_2_O_3_-2, was higher than that in the pristine N-Si alloy, which contained a small amount of residual Al from the manufacturing process. Furthermore, the Al content in Al_2_O_3_-2 was higher than that in Al_2_O_3_-1, possibly because of a longer TMA gas injection time. The XRF technique can only reveal the increase in the Al content of the samples. Thus, to verify the formation of the Al_2_O_3_ compound, XRD analysis of the samples was also performed.

### 3.2. XRD Patterns

**Figure 4:**
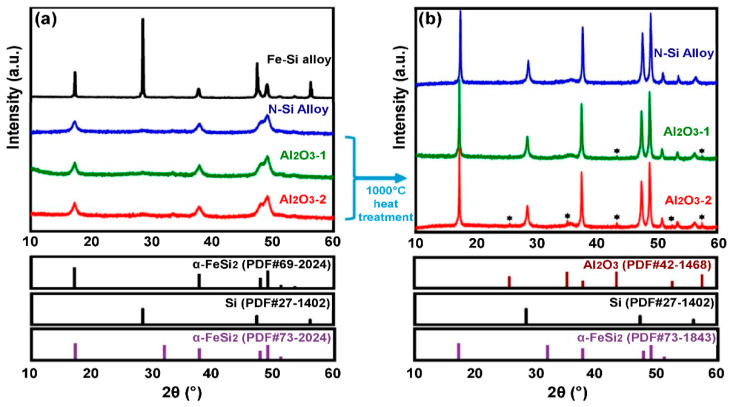
XRD patterns of the N-Si alloy, Al_2_O_3_-1, and Al_2_O_3_-2 powders: (**a**) Before heat treatment and (**b**) after heat treatment at 1000 °C. * new Peak was created (Al2O3-1,2, PDF#42-1468).

The change in the phase of the N-Si alloy, Al_2_O_3_-1, and Al_2_O_3_-2 powders were evaluated using XRD; the corresponding XRD patterns are shown in Figure 4. A pattern corresponding to Si (PDF#77-2107) and a sharp peak corresponding to α-FeSi_2_ (PDF#69-2024) are observed in the XRD pattern of the Fe–Si alloy powder (simple alloy) that was not machine milled (Figure 4a). The unmilled Fe–Si powder cannot be used as anode material because it is an alloy powder simply obtained by dissolving Fe and Si and then grinding the bulk. The XRD pattern of the mechanically milled N-Si alloy shows the peaks of Si (PDF#27-1402) and α-FeSi_2_ (PDF#73-1843). These peaks indicate that a milling energy sufficient to change the phase of Si to nanocrystals was applied because of the phase change of α-FeSi_2_; furthermore, a significant decrease in the intensity of the Si peak is also observed.

The XRD patterns of the ALD-synthesized Al_2_O_3_-coated powders, i.e., the Al_2_O_3_-1 and Al_2_O_3_-2, did not show the presence of an Al_2_O_3_ phase; instead, these patterns were the same as that of the N-Si alloy shown in Figure 4a. According to Shi et al. [52], if the Al_2_O_3_ layer is coated using the ALD process (using TMA and H_2_O as the precursors for Al and oxygen ions), then amorphous Al_2_O_3_ is formed. Furthermore, if the Al_2_O_3_ powder is heated alone at 900–1000 °C, then the α-alumina phase (PDF#73-1843) is generated, whose concentration increases with the increasing heat-treatment temperature [53]. To verify the existence of Al_2_O_3_ through XRD analysis, the N-Si alloy, Al_2_O_3_-1, and Al_2_O_3_-2 powders were heat-treated to 1000 °C for 3 h in an inert atmosphere at a heating rate of 10 °C/min. Figure 4b shows the XRD pattern of each powder heat-treated at 1000 °C. It can be seen that the peak intensities of the Si and α-FeSi_2_ phases increased significantly after the heat treatment. In addition, some phases were produced in Al_2_O_3_-1 and Al_2_O_3_-2, exhibiting diffraction peaks whose 2*θ* value coincides with that of the α-alumina phase (PDF#73-1843). This result indicates that amorphous Al_2_O_3_ was deposited on the powder during the ALD process, and Al was present as a compound of Al_2_O_3_, as evident from the XRF analysis.

Thus, according to the XRF and XRD analyses of the Al_2_O_3_ coating deposited by ALD, the concentration of the α-alumina phase in the Al_2_O_3_-2 powder, which was exposed to TMA gas for 0.5 s, was higher than that in the Al_2_O_3_-1 powder, exposed to TMA for 0.3 s. This result suggests that increasing the TMA exposure time increases the content and thickness of the Al_2_O_3_ layer. Next, Al_2_O_3_ was deposited as a layer on the powder surface using the ALD method, and BET analysis, S/TEM combined with energy-dispersive X-ray spectroscopy (S/TEM-EDS), and TEM analysis were conducted to assess the physical changes on the sample surface.

### 3.3. BET and TEM-EDS Analyses

**Figure 5:**
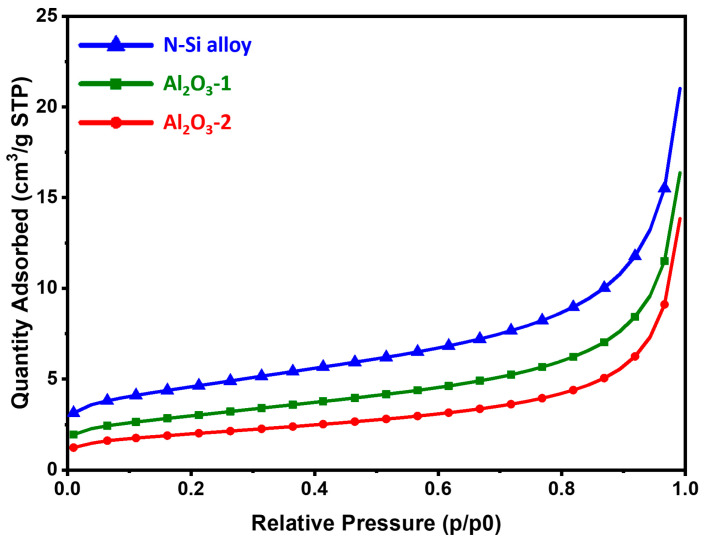
BET adsorption isotherms of the N-Si alloy, Al_2_O_3_-1, and Al_2_O_3_-2 powders.

The BET analysis was performed to characterize the physical properties of the samples through the specific surface area analysis of the powders. To improve the electrochemical properties of the anode powder, it is important to prevent side reactions with the electrolyte by securing an optimal (relatively low for the same volume of powder) surface area [54]. Table 4 shows the specific surface area, total pore volume, and average pore diameter of each powder. The results showed that the specific surface areas of the N-Si alloy, Al_2_O_3_-1, and Al_2_O_3_-2 powders decreased to 16.02, 10.52, and 7.03 m^2^/g. As the Al_2_O_3_ layer formed and its thickness increased, the total pore volume decreased, whereas the average pore diameter increased. This indicates that as the Al_2_O_3_ layer is formed and its content is increased, the narrow pores with a large volume changed to broad pores with a small pore volume [55]. Figure 5 shows the adsorption isotherm, which corresponds to a type II isotherm typically exhibited by non-porous or microporous absorbents [56,57]. The N-Si alloy powder without the Al_2_O_3_ layer showed a large absorption volume in the same relative pressure region (P/P_0_). Furthermore, Al_2_O_3_-2 showed a smaller absorption volume than that of Al_2_O_3_-1, suggesting a small pore volume on the surface. Therefore, a uniform coating layer of Al_2_O_3_ was formed, and as a result, the powder surface became relatively smooth, and the number of narrow pores on the surface decreased drastically [5,58,59]. As the specific surface area of the powder surface decreased, the area in contact with the electrolyte decreased as well. It is expected that this reduced contact can prevent the depletion of the electrolyte and deterioration of the electrochemical properties of the battery, thereby improving its performance [54,60,61,62].

**Figure 6:**
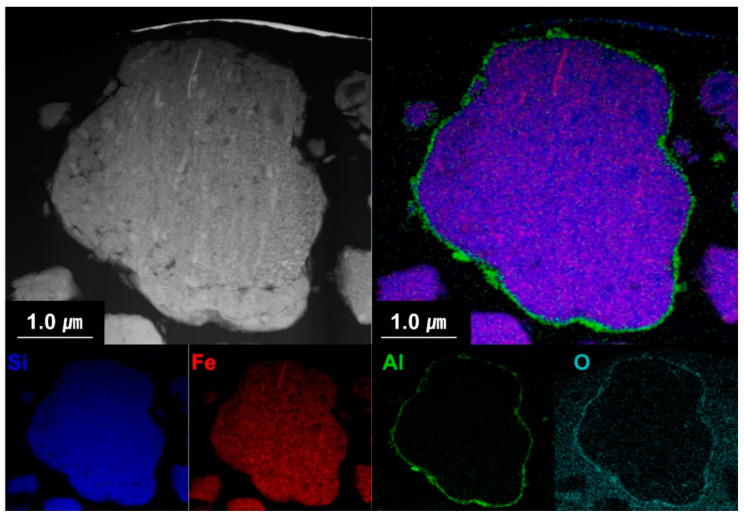
Cross-sectional S/TEM-EDS images of the Al_2_O_3_-2 powder.

**Figure 7:**
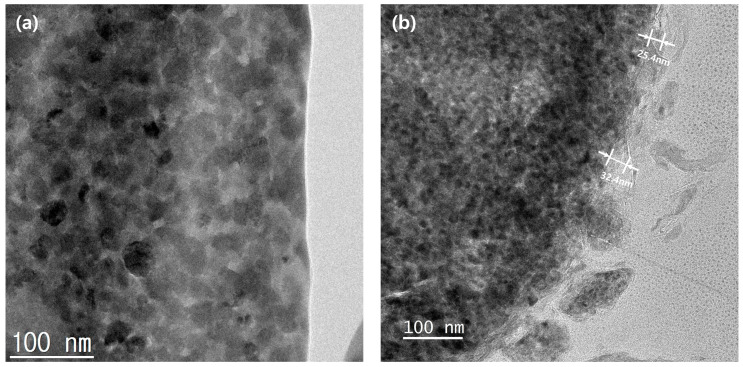
Cross-sectional TEM images of the powder surfaces: (**a**) N-Si alloy and (**b**) Al_2_O_3_-2.

Figure 6 shows the cross-sectional EDS mapping images of the Al_2_O_3_-2 powder after thinning by FIB milling. It can be seen that Al and O evenly wrap the powder surface. Figure 7 shows the TEM analysis results of the Al_2_O_3_-2 powder particles. No other materials are observed at the interface of the N-Si alloy powder. However, an amorphous layer with a thickness of ~30 nm without a lattice can be observed around the interface of the Al_2_O_3_-2 powder. This amorphous layer is confirmed as that of Al_2_O_3_, based on the XRD and S/TEM EDS results. The Al_2_O_3_ layer acts as a protective layer on the N-Si alloy. An effective electrochemical performance can be expected by reducing the cracks in the powders, caused by the continuous insertion and desorption of Li ions, as well as by suppressing the electrolyte reaction at the new interface that induces structural deterioration during the electrochemical reaction [5,59,61].

Next, the effect of the Al_2_O_3_ layer that evenly wraps the powders on the active anode material was investigated by evaluating their electrochemical properties.

### 3.4. Electrochemical Performance

**Figure 8:**
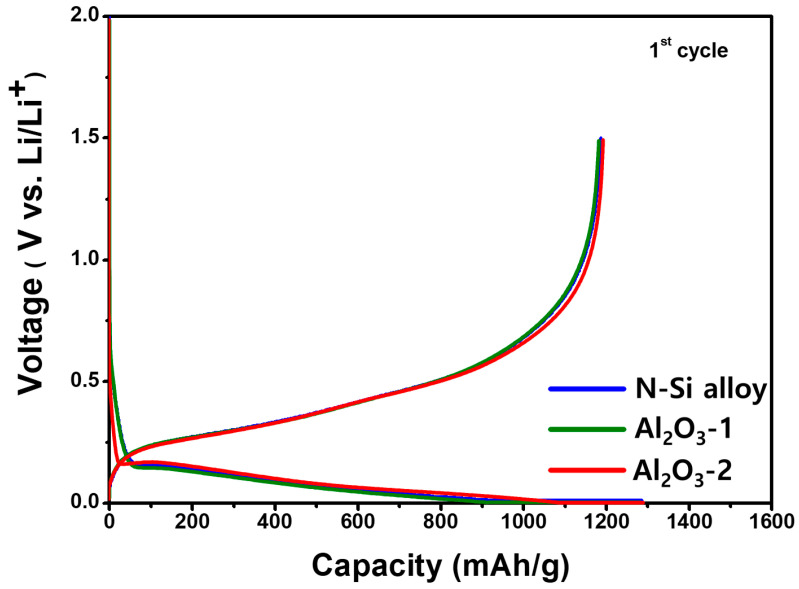
Potential curves of the CHCs, showing the first cycle for the N-Si alloy, Al_2_O_3_-1, and Al_2_O_3_-2 powders during lithiation/delithiation at a specific current of 100 mA g^−1^.

The initial capacity and Coulombic efficiency of the N-Si alloy, Al_2_O_3_-1, and Al_2_O_3_-2 powders were examined using CHCs fabricated with the PAA binder, a conductive material, and 80 wt.% of Si, followed by charging and discharging at a rate of 0.1 C; the corresponding results are shown in Figure 8. Table 5 shows the charge capacity, discharge capacity, and initial Coulombic efficiency (discharge capacity/charge capacity × 100) of the powders. Although a reduction in the specific capacity or decrease in the passivation of the Li ions was expected because of the formation of the Al_2_O_3_ layer, the discharge capacities of the N-Si alloy, Al_2_O_3_-1, and Al_2_O_3_-2 powders were 1186, 1184, and 1187 mAh/g^−1^, respectively, which are quite similar, regardless of the amount of Al_2_O_3_. This similarity implies an Al_2_O_3_ layer at these thicknesses does not decrease the specific capacity of the active material, nor does it affect the initial passivation of Li ions.

In addition, as the charging capacities of the N-Si alloy, Al_2_O_3_-1, and Al_2_O_3_-2 powders were similar, the thickness of the deposited Al_2_O_3_ layer did not reduce the specific capacity, implying that the Al_2_O_3_ partially reacted with the Li-ions to form a compound, Li_3.4_Al_2_O_3_ [63].

**Figure 9:**
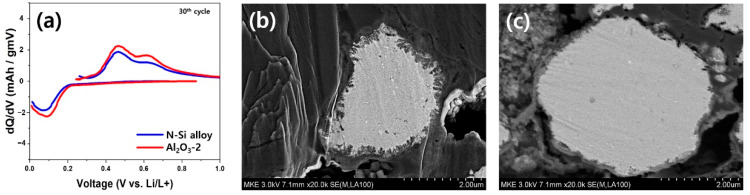
(**a**) Differential capacity plots of the N-Si alloy and Al_2_O_3_-2 powders, obtained after 30 charge/discharge cycles. SEM images of the (**b**) N-Si alloy and (**c**) Al_2_O_3_-2 powders after 30 charge/discharge cycles.

Figure 9 shows the dQ/dV curves of the CHC after 30 charge/discharge cycles; the CHC was assembled in the same way as described above, and the SEM images of particle cross-sections of the N-Si alloy and Al_2_O_3_-2 powders are also presented in Figure 9. The evaluation conditions are as follows. The protective layer was formed at a rate of 0.1 C for one charge/discharge cycle. Then, charging/discharging was conducted for 30 cycles at a rate of 1 C for one charge/discharge cycle. To verify the effect of the Al_2_O_3_ layer on the powder, the electrochemical behaviors of the powders during 30 charge/discharge cycles and the changes in the internal structure of the particles were analyzed. A delithiation peak was observed at approximately 0.05–0.2 V. The peak intensity of the cell fabricated with the N-Si alloy powders was lower than that of the cell prepared using Al_2_O_3_-2. Furthermore, the lithiation peak intensity of the N-Si alloy was also relatively low at 0.4–0.8 V. The result of particle cross-section analysis after 30 cycles showed that the powder surface deteriorated because of the continuous and excessive formation of side-reaction materials resulting from the direct contact between the powder surface with the electrolyte during the charge/discharge cycles. A thin layer of the side-reaction material (SEI layer) was formed on the surface of the Al_2_O_3_-2 powder, and the surface deterioration was less than that of the Si-alloy powder. This suggests that the ALD-synthesized Al_2_O_3_ layer prevented direct contact of the particles with the electrolyte during the charging/discharging, suppressing the continuous formation of the SEI layer [63,64]. The continuously and newly formed SEI layer degenerated the active interface of the anode powder and continuously consumed electrolytes, causing electrolyte depletion during long-term cycling [65,66,67].

**Figure 10:**
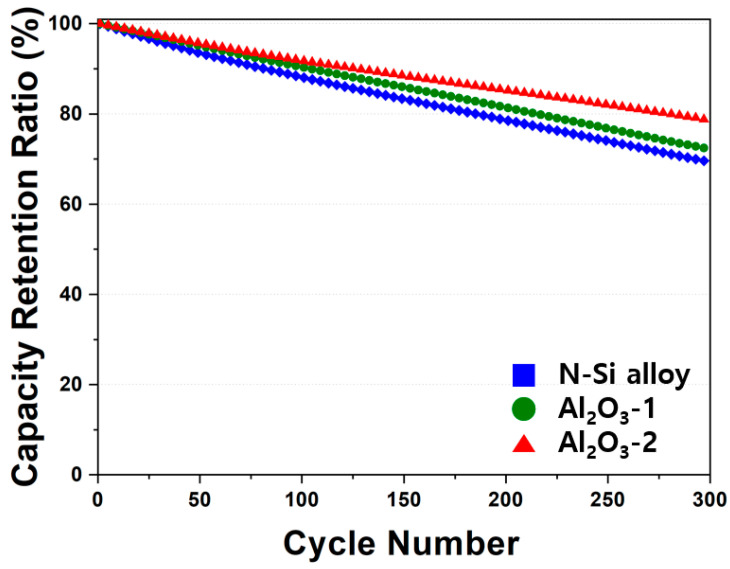
Cyclic performance of the N-Si alloy Al_2_O_3_-1 and Al_2_O_3_-2 powders.

The electrochemical behaviors of the powders were examined by assembling PFCs, whose lifespan and output characteristics were evaluated. Figure 10 shows that the N-Si alloy powders contained the largest number of irreversible areas, and the retention rate was improved from Al_2_O_3_-1 to Al_2_O_3_-2. This result suggests that the Al_2_O_3_ layer formed at the powder interface using the ALD technique prevented direct contact of the powders with the electrolyte as well as physical deterioration of the powders [63]. This suggests that the structural stability of the powders can be maintained during long-term cycling by preventing cracking and deterioration of the powders, as corroborated by the SEM analysis results of the powder cross-sections in the CHC (PAA binder), fabricated with a high ratio (80%) of Si active material, after 30 cycles. When the powders contract/expand according to the movement of Li ions, cracking of the powders is prevented by the Al_2_O_3_ layer that wraps the surface of the powder—a phenomenon not observed in the N-Si alloy [61]. 

**Figure 11:**
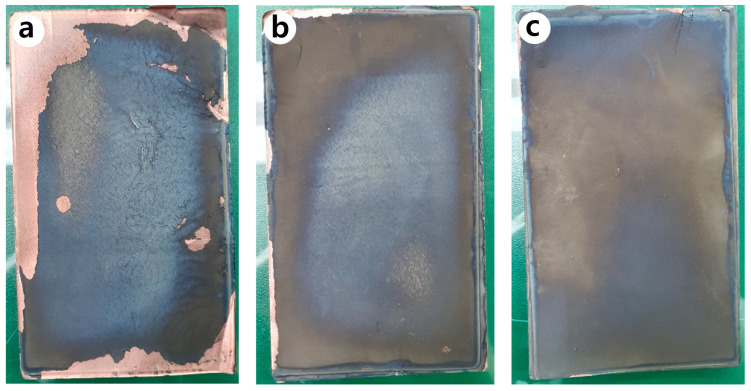
Photographs of the anodes inside the PFC, obtained after 600 cycles: (**a**) N-Si alloy, (**b**) Al_2_O_3_-1, and (**c**) Al_2_O_3_-2 powders.

**Figure 12:**
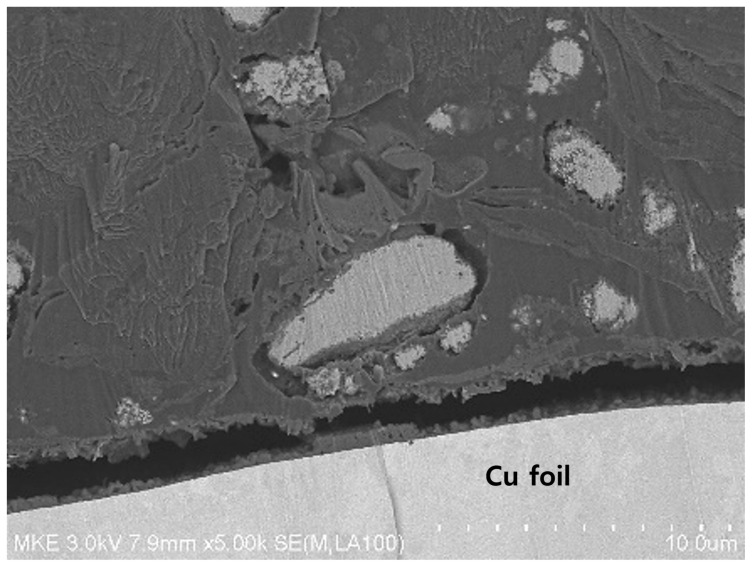
Cross-sectional SEM image of the N-Si alloy anode inside the PFC, obtained after 600 cycles.

Figure 11 shows the photographs of the anode of the jelly-roll sample developed by decomposing the PFC inside an inert glove box after 600 cycles. It can be seen that many of the anodes active elements present in the N-Si alloy were desorbed from the Cu foil, compared to the Al_2_O_3_-1 and Al_2_O_3_-2 powders. Figure 12 shows the SEM image of the cross-section of the N-Si alloy electrode plate (after 600 cycles). It can be seen that a space was generated between the N-Si alloy powder and graphite because of the contraction/expansion of the powders during the charge/discharge cycles. Furthermore, detachment from the Cu foil, represented by the overall swelling of the active anode material, can be observed. The pieces of active material detached from the electrode plates did not contribute to the electrochemical performance and possibly damaged the separator and electric short by acting as pollutants inside the pouch. The bonding between the powders and their adhesion with the Cu foil remained the same owing to the use of the same content of the SBR/CMC binder. This phenomenon was possibly caused by the expansion, cracking, and deterioration of the powders.

**Figure 13:**
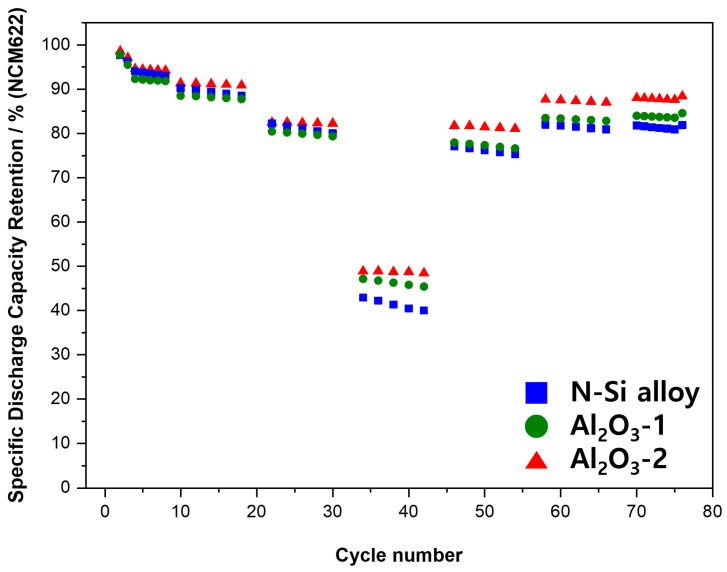
Results of the CC charging/discharging cycling experiments for the PFC at different C-rates.

The results of the rate performance evaluation of the PFCs are shown in Figure 13. The N-Si alloy showed the lowest performance at 3 C (high C-rate), whereas a performance degradation was observed from Al_2_O_3_-1 to Al_2_O_3_-2. After the high C-rate (3 C) operation N-Si alloy recovered 76% of its initial capacity retention rate at the subsequent low C-rate (2 C). This indicates that after the high C-rate (3 C) operation, the powders were physically deteriorated and were unable to recover their capacity retention rates observed at lower C-rates. Furthermore, although Al_2_O_3_-1 showed good capacity retention at 3 C (high C-rate), its capacity recovery (at 2, 1, and 0.5 C) was similar to that of the N-Si alloy. This indicates that although the thinner Al_2_O_3_ layer in Al_2_O_3_-1 showed a relatively more stable retention rate (at 0.5 or 1 C) compared to that of the Al_2_O_3_-2 powder, the deterioration of the alloy powders was higher than that of the Al_2_O_3_-2 powder at 3 C (high C-rate). It is considered that the existence of a uniform layer with an appropriate thickness on the powder surface enhances the durability and electrochemical stability of these powders during high C-rate operations [63]. 

**Figure 14:**
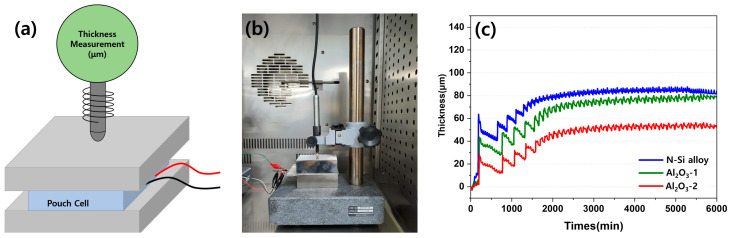
(**a**) Schematic diagram of the setup for detecting SLPC volume expansion and contraction. (**b**) Photograph of the equipment for detecting SLPC volume expansion and contraction behavior. (**c**) Volume expansion–contraction graph of each SLPC during long-term charge/discharge cycling.

The contraction and expansion behaviors of the pouch cell during the charging and discharging were observed using an SLPC. Figure 14 shows a schematic of the equipment used to measure the volume expansion and contraction of the SLPC, along with the photograph of the equipment used in this study. The fine expansion and contraction during the charging and discharging of the SLPC, respectively, were detected using a digital thickness gauge and plotted in the form of electric signals. One kilogram of cuboid metals was placed above and below the SLPC to ensure an even distribution of the electrolyte inside the pouch and on the anodes to measure the overall thickness changes. The thickness was measured over time (minutes) starting from the formation cycle, and the amplitude of the signal indicates changes in the thickness for each cycle during long-term cycling (charging, discharging) in the graph in Figure 14c.

Evidently, the SLPC containing the N-Si alloy anode showed a larger increase in thickness during the first charging cycle than those containing the Al_2_O_3_-1 and Al_2_O_3_-2 powders. After the formation stage, the contraction and expansion became stable; however, the Al_2_O_3_-2 and N-Si alloy powders better maintained their thicknesses (approximately 50 and 80 μm, respectively). During the initial rapid expansion period, the expansion rate of the N-Si alloy increased more than the contraction rate. Al_2_O_3_-1 and Al_2_O_3_-2 also underwent contraction and expansion, but they expanded to a lower degree in the beginning compared to the N-Si alloy; in addition, the initial expansion rate of Al_2_O_3_-1 was higher than that of Al_2_O_3_-2. Furthermore, the slopes of both Al_2_O_3_-1 curves rise steeply and continuously for 2000 min and indicate a thickness similar to that of the N-Si alloy. This result indicates that the Al_2_O_3_-1 layer was thinner than the Al_2_O_3_-2 layer and better suppressed the expansion at the beginning. However, the surface of the powder deteriorated as the charging and discharging continued. Consequently, the thickness of the pouch cell became similar to that of the N-Si alloy during long-term cycling. 

## 4. Conclusions

In this study, we employed the ALD technique to deposit Al_2_O_3_ layers on the surface of N-Si alloy powders fabricated using a mechanical alloying process. To analyze the amorphous Al_2_O_3_ structure, the powders were heat-treated at 1000 °C. The crystallized Al_2_O_3_ was examined using XRD patterns, which showed that the amount of Al_2_O_3_ deposited on the powders increased with the increasing TMA exposure time, and the formation of a uniform layer on the powder surface was confirmed through particle size and S/TEM-EDS analyses of the powders.

In addition, the effect of the specific surface area on the layer formation was examined by analyzing the electrochemical behaviors. The thickness of the Al_2_O_3_ layer evenly formed on the powder surface did not influence the initial capacity and Coulombic efficiency of the powders. The PFCs with pristine N-Si alloy powder retained 69.8% of their original capacity after 300 cycles, but when the powder was coated with Al_2_O_3_, this capacity retention improved to 72.3% and 79.1% for Al_2_O_3_-1 and Al_2_O_3_-2, respectively. Furthermore, the cross-sectional structural analysis of the powders revealed that the deterioration of the N-Si alloy powders was effectively prevented by the Al_2_O_3_ layer. This layer prevents direct contact with the electrolyte, thereby suppressing the formation of the SEI layer, as verified by CHCs. In addition, the Al_2_O_3_ layer suppresses the expansion of powder during charging and discharging, which in turn prevents the anode powder from detaching from the graphite and the copper plate, as confirmed in PFCs and SLPCs.

The analysis of the PFC anodes after 600 cycles indicated that the formation of the Al_2_O_3_ layer improved the cycle-life retention rate by preventing desorption of the Cu foil and anode material. This suggests that the Al_2_O_3_ layer prevented direct contact between the powders and the electrolyte, thereby suppressing the formation of SEIs and preventing the depletion of the electrolyte. Furthermore, the expansion of the N-Si Alloy powders was suppressed because of the reduced expansion/contraction of the pouch during the charging/discharging of the SLPC.

The results obtained in this study verify that the electrochemical properties of N-Si alloy powders can be improved by forming a protective layer on the powder surface using the ALD technique, which can be commercialized owing to its low-temperature stability and simple processing. Thus, the performance of an LIB containing commercial Si-based anodes can be enhanced using this ALD coating method.

## Figures and Tables

**Table 1:** Processing times for the different process steps and conditions for the ALD experiment.

ALD Process Conditions (1 Cycle)							
Process		Injection of TMA Gas	Soaking Time	Injection of N_2_ Gas	Injection of H_2_O Gas	Soaking Time	Injection of N_2_ Gas
**Time**	Al_2_O_3_-1	0.3 s	20 s	30 s	0.3 s	20 s	30 s
	Al_2_O_3_-2	0.5 s	20 s	30 s	0.3 s	20 s	30 s

**Table 2:** C-rate conditions set for the evaluation of rate performance characteristics.

Evaluation Conditions		
C-Rate	Number of Charge/Discharge Cycles	Classification
0.1 C	1 cycle	Acceleration
0.2 C	1 cycle	
0.5 C	5 cycles	
1.0 C	5 cycles	
2.0 C	5 cycles	
3.0 C	5 cycles	
2.0 C	5 cycles	Recovery
1.0 C	5 cycles	
0.5 C	5 cycles	
0.2 C	1 cycle	
0.1 C	1 cycle	

* 0.2 C charge/discharge every cycle at 1–3 C in output and recovery (not shown in graph).

**Table 3:** Composition of each powder sample as evaluated by XRF.

wt.%	Al	Si	Fe	Mn	Cr
N-Si alloy	0.1	71.8	23.9	3.9	0.3
Al_2_O_3_-1	0.8	71.7	23.4	3.8	0.3
Al_2_O_3_-2	1.6	71.7	22.6	3.8	0.3

**Table 4:** BET surface area, total pore volume, and average pore diameter of the N-Si alloy, Al_2_O_3_-1, and Al_2_O_3_-2 powders.

Sample	BET Surface Area (m^2^/g)	Total Pore Volume (cm^3^/g)	Average Pore Diameter (Å)
N-Si alloy	16.02	0.032	81.2
Al_2_O_3_-1	10.52	0.025	96.3
Al_2_O_3_-2	7.03	0.021	121.7

**Table 5:** Electrochemical parameters of the CHCs: charge capacity, discharge capacity, and initial Coulombic efficiency of the N-Si alloy, Al_2_O_3_-1, and Al_2_O_3_-2 powders.

	N-Si Alloy	Al_2_O_3_-1	Al_2_O_3_-2
Charge capacity (mAh/g^−1^)	1286	1286	1283
Discharge capacity (mAh/g^−^^1^)	1186	1184	1187
Initial Coulombic efficiency (%)	92.2	92.1	92.5

## Data Availability

Not applicable.
